# Pharmacokinetic/pharmacodynamic modeling for the determination of a cimicoxib dosing regimen in the dog

**DOI:** 10.1186/1746-6148-9-250

**Published:** 2013-12-11

**Authors:** Elisabeth C Jeunesse, Marc Schneider, Frederique Woehrle, Mathieu Faucher, Herve P Lefebvre, Pierre-Louis Toutain

**Affiliations:** 1 INRA, UMR1331 Toxalim, F-31076 Toulouse, France; 2 Unité de Recherche Clinique et Département des Sciences Cliniques, Université de Toulouse, INP, Ecole Nationale Vétérinaire de Toulouse, Toulouse, France; 3 Vétoquinol Research and Development, Lure, France; Vétoquinol S.A., rue des Jeûneurs, Paris, France; 4 Université de Toulouse, INP, Ecole Nationale Veterinaire de Toulouse, Toxalim, F-31076 Toulouse, France

**Keywords:** Pharmacokinetic, Pharmacodynamic, Cimicoxib, NSAID, Dog, Dosage regimen

## Abstract

**Background:**

Cimicoxib is a new coxib anti-inflammatory drug for use in the dog. To determine a preclinical dosage regimen for cimicoxib in dog, a reversible model of kaolin–induced paw inflammation was used. Dosage regimens were established using pharmacokinetic/pharmacodynamic (PK/PD) modeling approach (indirect response model).

**Results:**

Analgesic, anti-inflammatory and antipyretic endpoints investigated with the inflammation model established the efficacy of cimicoxib at a dose of 2 mg/kg administered orally (single dose) in 12 beagle dogs.

For both the oral and IV route of administration two groups of dogs to be identified namely Poor Metabolizers (PM) and Extensive Metabolizers (EM).The terminal half-life after oral administration was 8.0 ± 0.6 h for the PM and 4.6 ± 2.6 h for the EM groups, with the corresponding values after the IV route being 5.6 ± 1.7 h and 2.7 ± 0.9 h (mean ± SD).

The main pharmacodynamic parameters (potency, efficacy, and sensitivity) were estimated for four endpoints (body temperature, creeping speed, ground vertical reaction force and clinical lameness score). The plasma concentration corresponding to half the maximum of the indirect effect were 239 μg/L for creeping speed, 284 μg/L for the lameness score, 161 μg/L for the ground reaction vertical force and 193 μg/L for the body temperature.

To document possible polymorphism of the cimicoxib disposition in the target dog population, cimicoxib was administered by the intravenous route to 40 dogs (four different sized breeds). The cimicoxib half-lives in these 40 dogs were of same order of the magnitude as those of the EM beagle dogs. Thus pharmacokinetic and pharmacodynamic parameters obtained from the EM beagle dogs were selected to simulate the dose-effect relationship of cimicoxib after an oral administration allowing a dosage regimen to be selected for confirmation by a clinical trial.

**Conclusions:**

Cimicoxib was an efficacious anti-inflammatory, antipyretic and analgesic drug and a dosage regimen of 2 mg/kg daily was determined for confirmatory clinical trials.

## Background

Non-Steroidal Anti-Inflammatory Drugs (NSAIDs) constitute an essential therapeutic class both in human and veterinary medicines. It is now well established that most side effects of NSAIDs are related to their anti-cyclooxygenase-1 (anti-COX-1) activity while the therapeutic effects are mainly associated with COX-2 inhibition. This has led to the development of preferential or selective COX-2 inhibitors among which are the diarylimidazol derivatives (COXIB class) in an attempt to overcome the side effects especially those of the gastro-intestinal tract (g.i.t). One of these substances was recently developed for therapeutic use in dogs: cimicoxib which is a sulfonamide, like celecoxib, mavacoxib and firocoxib. High in vitro potency of cimicoxib was measured for COX-2 inhibition using a human whole blood assay (half maximal Inhibitory Concentration (IC50) 66nM or 25 ng/mL). In vivo effects were demonstrated in rats in adjuvant-induced arthritis, and in air pouch and hyperalgesia models [1]. In fact, the COXIB class is not homogenous regarding the COX-1/COX-2 selectivity and some of its members such as deracoxib and mavacoxib are classified as preferential and moderately selective COX-2 inhibitors while others like firocoxib and robenacoxib are classified as highly selective COX-2 inhibitors with a more than 100-fold greater potency for COX-2 inhibition and with little or no *in vivo* inhibition of COX-1 [2]. Usually, with highly selective COXIB compounds, no gastro intestinal tract (g.i.t) ulceration or antiplatelet effects are expected even at a maximum plasma concentration while significant COX-1 inhibition may occur with preferential COXIBs [2]. For example, for deracoxib, the COX-1/COX-2 selectivity index (using a whole blood assay and expressing results as the ratio of IC_50_) was about 50–60 [3] and gastric ulcerations were reported following some overdosing of deracoxib or as a result of its association with other substances such as other NSAIDs or corticosteroids [4,5]. This means that a COX-1/COX-2 selectivity index of 50 is not enough to guarantee a margin of g.i.t safety and more highly selective COXIBs are desirable. This is the case of firocoxib with a selectivity index of 384 in a whole blood assay [6] and for which a favorable tolerability was shown in a large scale survey with a withdrawal rate associated with g.i.t side effects as low as 2.9% of dogs (mainly vomiting) and no serious drug-related adverse events [7]. However it was shown that firocoxib was able to slow down wound healing in a canine gastric mucosal injury model but by a mechanism independent of prostaglandin synthesis [8] meaning that a high COX-1/COX2 selectivity is not the only pharmacodynamic (PD) endpoint to consider when discussing NSAID g.i.t tolerability. Another factor of COXIB tolerability is the selected dosage regimen; a dosage regimen depends on two pharmacokinetic (PK) parameters (namely plasma clearance and bioavailability for the extravascular route of administration) and of one PD parameter (namely the efficacious plasma concentration that reflects the drug potency). In addition, the dosage interval should also be rationally determined according to the plasma terminal half-life (which itself depends on plasma clearance and volume of distribution [9]). For the COXIB class, the plasma clearances are very different among substances from a very low clearance for mavacoxib (2.7 mL/kg/h) [10] to a rather high clearance for robenacoxib (810 mL/kg/h) [11] and firocoxib (462 ml/kg/h) [6] which explains the large differences in half-lives namely 17.3 days (beagle dogs) for mavacoxib, 0.63 h (beagle dogs) for robenacoxib and 5.9 h (mixed-breed dogs) for firocoxib. Such differences are reflected into the dosage regimen (dose and dosing interval), with mavacoxib marketed at 2 mg/kg at one month intervals *vs.* 5 mg/kg daily for firocoxib and 1-2 mg/kg daily for robenacoxib. It is expected that a very short half-life of about 1 h cannot maintain steady plasma concentrations over the entire daily dosage interval while a very long terminal half-life is ineluctably associated with a minimal delay (about 3–4 times the duration of half-life) to achieve steady state conditions. In addition a population PK survey for mavacoxib in osteoarthritic dogs, showed wide between-subject variability with a typical terminal half-life (population mean) of 44 days, but for some dogs (5% of the population) it exceeded 80 days with extreme values up to 140 days. It was shown for mavacoxib that the PK differed considerably between young adult beagle dogs and the typical geriatric large-breed osteoarthritic patient [12] indicating that considering only preclinical PK in young beagle dogs can be very misleading when rationally determining a dosage regimen for a COXIB. In the present paper this point was specifically addressed for cimicoxib by investigating the PK profiles in breeds other than beagle dogs.

The present study reports how the dosage regimen of cimicoxib (Cimalgex ND) was established using PK/PD concepts [13-22] and provides preliminary information on the interbreed variability of cimicoxib PK.

## Results

### Study 1

**Figure 1:**
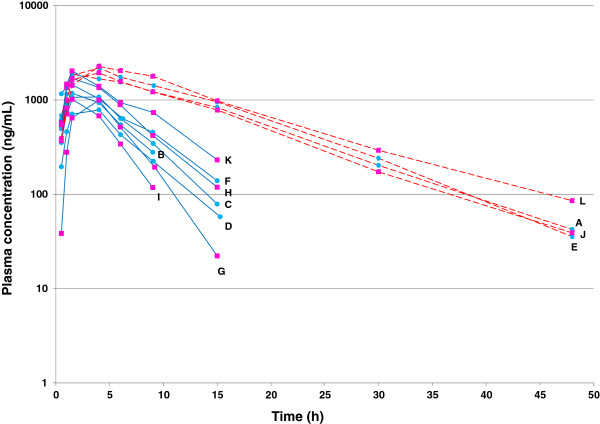
**Individual plasma concentrations of cimicoxib (ng/mL) *vs. *time (h) after a single oral administration of cimicoxib (2 mg/kg) in 12 beagle dogs.** Visual inspection of the figure suggests the existence of 2 subgroups of dogs which were named “Poor Metabolizers” (red dotted line) and “Extensive Metabolizers” (blue continuous line) (see the text for explanation). Pink squares were female dogs and blue circles male dogs. Letters indicates names of dogs.

**Figure 2:**
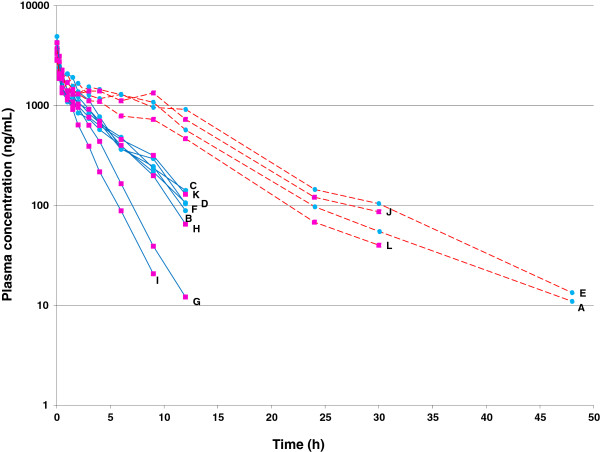
**Individual plasma concentrations of cimicoxib (ng/mL) *vs. *time (h) after a single intravenous administration of cimicoxib (2 mg/kg) in 12 beagle dogs.** Visual inspection of the figure confirms what has been observed in Figure 1 with the existence of the same 2 subgroups of dogs named “Poor Metabolizers” (red dotted line) and “Extensive Metabolizers” (blue continuous line). Letters indicates names of dogs.

The time course of cimicoxib plasma concentrations *vs.* time (h) following oral administration is presented in Figure 1. Visual inspection of the graph suggests the existence of two subgroups of dogs regarding the terminal half-life, with 4 dogs having a long terminal half-life (8.0 ± 0.6 h) while the 8 other dogs exhibited a short terminal half-life (2.9 ± 0.9 h) p < 0.0001). Plasma terminal half-life is a hybrid parameter controlled by plasma clearance and volume of distribution and the difference in the rate of elimination between the two groups of dogs was suggestive of a difference in their capacity to metabolize the cimicoxib i.e. of a difference in plasma clearance. To verify this hypothesis, the PK parameters of cimicoxib were also estimated for the same twelve dogs after a single intravenous (IV) cimicoxib administration (Figure 2). The main IV PK parameters are summarized in Table 1 and showed a significant difference between the two groups of dogs for cimicoxib half-life (2-fold) and clearance (3-fold) while the volumes of distribution were not significantly different (p > 0.05). By analogy to the terminology adopted for celecoxib [23], another COXIB for which a bimodal repartition of a beagle dog population was observed, we designated the group of beagle dogs rapidly eliminating cimicoxib as “Extensive Metabolizers (EM)” and the group of dogs slowly eliminating cimicoxib as “Poor Metabolizers (PM)”, even if the cimicoxib metabolism *per se* was not directly investigated in the present experimentation.

**Table 1:** Pharmacokinetic parameters (mean ± SD) of cimicoxib administered by the IV route at 2 mg/kg to 12 dogs

**Parameters**	**Extensive metabolizers (n = 8)**	**Poor metabolizers (n = 4)**
**AUC (h.μg/L)**	7202 ± 1814*	19151 ± 3149*
**V (L/kg)**	1.12 ± 0.203	0.89 ± 0.133
**HL (h)**	2.72 ± 0.858*	5.63 ± 0.727*
**CL (L/h/kg)**	0.31 ± 0.118*	0.11 ± 0.022*

Data were analysed using non-compartmental approach; AUC: Area under the curve; V: Volume of distribution; HL: Plasma half- life; Cl: plasma clearance.

^*^Difference were significant between the two groups p < 0.001 for HL and AUC and p < 0.01 for the CL.

**Figure 3:**
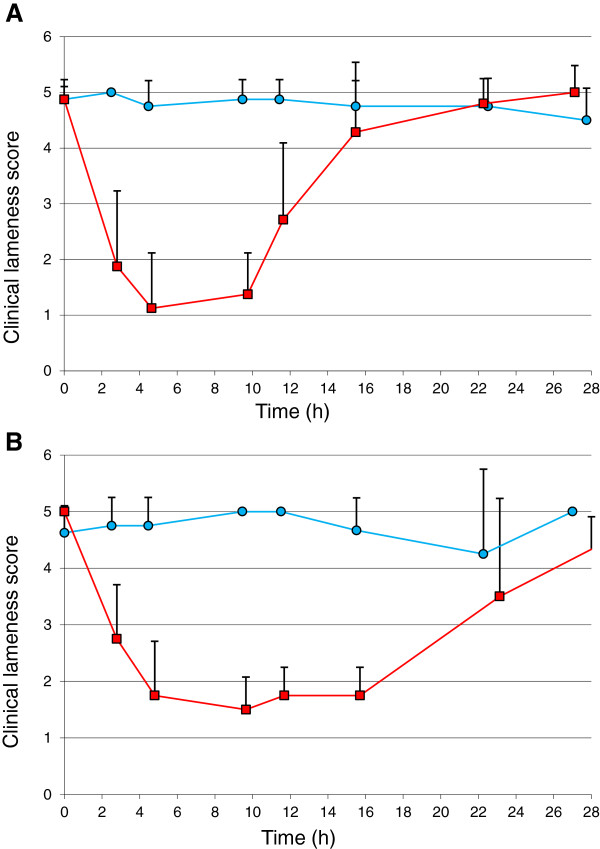
**Improvement of the clinical lameness score observed after an oral administration of cimicoxib vs.placebo in PM and EM dogs.** Improvement of the clinical lameness score was observed (mean score + SD) *vs.* time (h) after oral administration of cimicoxib at 2 mg/kg (red squares) or placebo (blue circles) after induction of paw inflammation in the 8 Extensive metabolizer (EM) dogs **(A)** and in 4 Poor metabolizer (PM) dogs **(B)**. The same maximal effect was observed for the two groups of dogs but the duration of the effect was longer for the PM than for the EM group reflecting the different cimicoxib disposition observed between these two groups of dogs (see Figure 1). 0 no lameness; 1 slight lameness: barely perceptible throughout almost the whole observation period; 2 obvious lameness but the animal maintains its limb on the ground; 3 moderate lameness, the animal rests the limb on the ground slightly; 4 severe lameness: the animal uses its limb (makes contact with and/or skims the ground) but it does not put its weight on the limb; 5 maximum lameness: the animal refuses to move (it remains sitting or lying down) and/or avoids putting limb on the ground at all.

**Figure 4:**
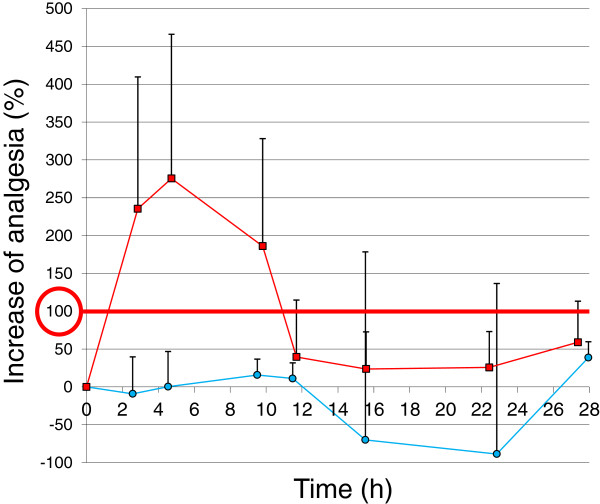
**Time development of the analgesic effect after an oral administration of cimicoxib at 2 mg/kg vs. placebo administration in 8 extensive metabolizer dogs.** Pain was assessed using a hind paw thermal escape model in control conditions (no inflammation) and after a kaolin injection in the paw. The paw withdrawal time was measured in seconds and expressed as a percentage; 100% is the control value without inflammation and 0% (at time 0) is the control value after induction of inflammation. Visual inspection of the figure shows that cimicoxib can develop an analgesia that is higher than 100% i.e. that under cimicoxib, the withdrawal time of the inflamed paw was longer than in control condition without inflammation. This suggests that the analgesic effect of cimicoxib is not only due to its anti-inflammatory effect but also to an intrinsic analgesic property.

For all clinical endpoints an improvement after cimicoxib administration was observed for the EM and the PM dogs. For the six pharmacodynamics endpoints investigated (excepting the lameness score), the maximum effect was expressed as a percentage of improvement, a 100% effect indicating that the measures returned to control values (reference measure before inflammation) and a 0% effect indicating a complete lack of effect of the cimicoxib. For the clinical lameness score, the effects were expressed using a score ranging from 0 (no lameness) to 5 (5 maximum lameness). For each endpoint, the amplitude and duration of the maximum effect were described for each of the two groups of dogs (Table 2). The maximum effect on skin temperature could not be evaluated in the Poor-elimination group because no plateau could be observed, with the improvement increasing constantly for this endpoint from 37% to 91%. For the other endpoints, the maximal observed effect was similar for the two groups of dogs but the duration of a sustained maximal effect and the duration of the overall effect were systematically longer in the group of PM dogs (see Figure 3). The overall maximal effects for the two groups of dogs were 86% for the body temperature, 66% for the ground reaction vertical force and 76% for the creeping speed. In contrast, the corresponding duration of effect for these endpoints was systematically longer in the PM group (from 25 to 28 h for the PM group *vs*. from 13 to 16 h for the EM group) (see Table 2 for details). For the analgesic effect of cimicoxib, it was observed that the tolerance threshold to the heat-induced pain was increased above those recorded under control conditions (i.e.100%) to reach a maximum of 232 ± 45% for the EM group and 152 ± 53% for the PM group (Figure 4) which is indicative of a cimicoxib analgesic effect that is not limited to an anti-inflammatory effect (see Discussion).

**Table 2:** Level and duration of maximum improvement for each endpoint describing the effects of the cimicoxib (2 mg/kg) administered by the oral route in the 4 Poor Metabolizers (PM) and 8 Extensive Metabolizers (EM) dogs

**Endpoint**	**Maximum effect**		**Duration of the maximal effect**		**Total duration of the effect**	
	**PM**	**EM**	**PM**	**EM**	**PM**	**EM**
**Body temperature**	87 ± 9%	84 ± 8%	19.6 h (from 2.1 to 21.7)	7 h (from 2.1 to 9.1 h)	25 h	13 h
**Paw circumference**	15 ± 3%	14 ± 2%	7 h (from 4.2 to 11.2 h)	9 h (from 2.2 to 11.2 h)	20 h	15 h
**Skin plantar temperature**	No plateau observed	38 ± 6%	Could not be determined	7 h (from 2.3 to 9.3 h)	22 h	13 h
**Creeping speed**	77 ± 7%	76 ± 9%	11.1 h (from 4.2 to 15.3 h)	7 h (from 2.2 to 9.2 h)	25 h	15 h
**Clinical lameness score**	1 or 2	1 or 2	10.9 h (from 4.8 to 15.7 h)	8.8 h (from 2.8 to 16 h)	not assessed	not assessed
**Vertical normalized force**	67 ± 6%	68 ± 7%	11.1 h (from 4.5 to 15.5 h)	6.9 h (from 2.6 to 9.5 h)	28 h	16 h
**Analgesia**	162 ± 53%	232 ± 45%	8.8 h (from 2.9 to 11.8 h)	7 h (from 2.9 to 9.8 h)	23 h	not assessed

For the body temperature, the paw circumference, the creeping speed, the vertical normalised force obtained with a force plate and the analgesic effect, the maximal estimated effect was expressed as a percentage of control value, a 100% maximum effect indicating a return to control value (no kaolin challenge). Analgesia was assessed by the paw withdrawal time to a heat stimulus and values higher than 100% indicate that there was an analgesic effect of cimicoxib independent of its anti-inflammatory effect i.e. that the paw withdrawal time was longer after the cimicoxib administration than in control condition (no inflammation). For the lameness, the clinical score ranged from 0 (no lameness) to 5 (maximal lameness): a score of 1 corresponding to a slight lameness and a score of 2 to an obvious lameness. The duration of the effect (maximal or total) was directly obtained from raw data. Duration of total effect is the time when the effects of cimicoxib observed are superior to the placebo. Duration of maximal effect is duration during which a steady maximal effect was observed. Values are mean ± SD for the maximal effect and mean and range value for the duration of the maximum effect.

**Figure 5:**
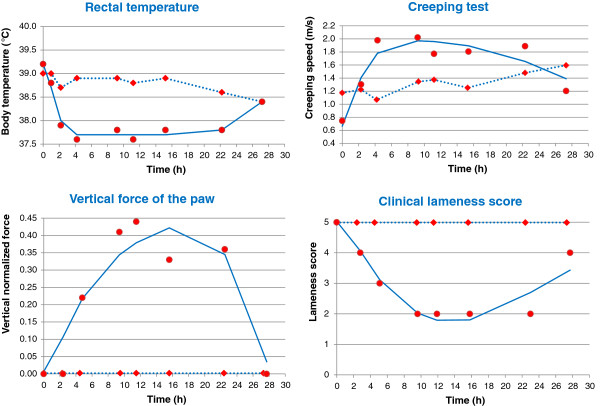
**Observed ( ● ) and PK/PD predicted (-) effects of cimicoxib *vs. *time (h) after an oral administration of cimicoxib at 2 mg/kg and observed ( ♦, ●●● ) effects of placebo *vs. *time (h) for 4 endpoints in a representative poor metabolizer dog.** Predicted PK/PD effects were obtain by fitting PD data using an indirect effect model (see Methods for details), for lameness score from 0 (no lameness) to 5 (maximal lameness), see Figure 3 for a definition of the different scoring.

Figure 5 shows the observed and fitted values of the four modelled endpoints for a representative dog. The efficacy and potency parameters obtained for the four modelled pharmacodynamic endpoints are presented in Table 3. Within a given group, the EC_50_/IC_50_ (half maximum effective plasma concentration) values were relatively similar, ranging from 325 ng/ml for the ground reaction vertical force to 786 ng/ml for the lameness for the group of PM dogs but from 161 to 284 ng/mL for the EM dogs (p < 0.001).

**Table 3:** Pharmacodynamics parameters describing the cimicoxib effect after an oral administration of cimicoxib (2 mg/kg) in 4 poor metabolizers (PM) and 8 extensive metabolizers (EM) dogs

	**EC**_**50 **_**or IC**_**50 **_**(ng/mL)**		**Emax (no unit)**		**Imax (no unit)**		**Kin (°C.h**^**-1 **^**or m.s**^**-1**^**.h**^**-1 **^**or score.h**^**-1 **^**or normalised force.h**^**-1**^**)**		**Kout (h**^**-1**^**)**		**n (no unit)**	
	**PM**	**EM**	**PM**	**EM**	**PM**	**EM**	**PM**	**EM**	**PM**	**EM**	**PM**	**EM**
**Body temperature (°C)**	473.4 [422.7, 558.9]	193.2 [95.9, 330.0]	0.04 [0.03, 0.04]	0.04 [0.03, 0.06]	N.A	N.A	45.84 [42.76, 49.19]	41.30 [20.76, 84.13]	1.17 [1.09, 1.25]	1.04 [0.53, 2.10]	6.99 [1.99, 10.00]	5.09 [1.31, 10.00]
**Creeping speed (m/s)**	344.5 [140.0, 592.7]	239.4 [136.0, 427.5]	N.A	N.A	0.70 [0.44, 0.87]	0.71 [0.38, 1.00]	0.76 [0.36, 0.97]	0.90 [0.15, 2.71]	1.07 [0.80, 1.47]	0.95 [0.24, 2.02]	3.68 [0.49, 10.00]	4.74 [0.71, 10.00]
**Lameness score (no unit)**	785.8 [579.4, 916.1]	284.3 [71.1, 827.8]	N.A	N.A	Fixed at 1	Fixed at 1	2.57 [0.70, 4.96]	5.34 [1.17, 16.87]	0.509 [0.14, 0.99]	1.08 [0.22, 3.54]	2.70 [0.66, 5.03]	4.25 [1.12, 10.00]
**Vertical force (no unit)**	325.1 [235.3, 397.6]	160.8 [51.4, 339.3]	N.A	N.A	0.97 [0.95, 0.99]	0.98 [0.93, 1.00]	0.09 [0.07, 0.10]	0.09 [0.06, 0.10]	8.47 [5.09, 9.99]	7.40 [3.21, 10.00]	5.67 [4.21, 6.67]	6.13 [2.13, 9.73]

Parameters were estimated using indirect effect PK/PD models. Mean and range, Emax: maximum effect, EC_50_: plasma concentration to obtain 50% of the Emax, IC_50_: plasma concentration to obtain 50% of the Imax, Kin: the zero-order rate constant for the production of the response; Kout: the first-order rate constant for the loss of the response; n: coefficient of Hill that measures the slope of the concentration-effect relationship; N.A.: Not Applicable.

**Figure 6:**
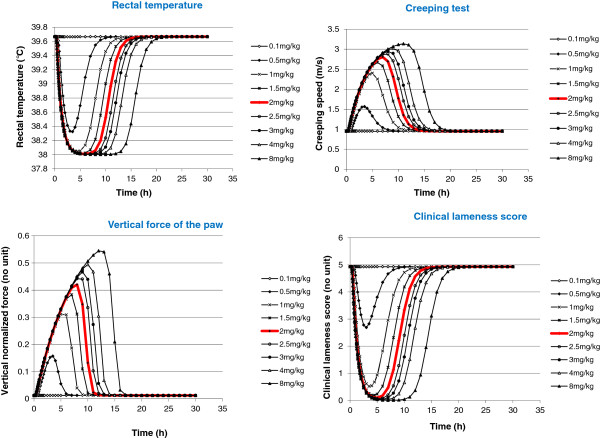
**Simulated values of the effects of the cimicoxib for different oral doses of cimicoxib for the 4 endpoints modeled using a PK/PD model in the 8 extensive metabolizer dogs.** Effects of cimicoxib *vs.* time (h) were simulated using the corresponding PK/PD model for different oral doses of cimicoxib (from 0.1 to 8 mg/kg) on the rectal temperature (°C), the normalized vertical ground reaction force of the paw, the creeping speed (m/s) and the overall clinical lameness score. PK and PD parameters used for simulations were the means of those obtained in the 8 extensive metabolizer dogs. The thick line was obtained with 2 mg/kg, the dose that has been selected for confirmatory clinical trials. Beyond this 2 mg/kg dose, the effects were not or marginally increased: the only consequence of the increase in the dose being to increase the duration of the effect.

The average PK and PD parameters obtained in the 8 dogs of the EM subgroup (see Discussion) were used to simulate the plasma concentration profiles and the effects predicted by the PK/PD model of different dosing regimens of cimicoxib administered by the oral route (from 0.1 to 8 mg/kg). For this simulation, a dose-linearity of the PK parameters was assumed but after the completion and submission of this work, we have had access to an unpublished experimental trial showing that the disposition of cimicoxib after an oral administration was not linear for doses ranging from 1 to 4 mg/kg due to the limited solubility of cimicoxib (Vetoquinol, unpublished results); for a dose of 1 mg/kg, the plasma concentrations were higher than expected (corresponding to a dose of 1.46 mg/kg for our simulations) and for the dose of 4 mg/kg, the plasma concentrations were lower than expected corresponding to a dose of 3.18 mg/kg for our simulations; nevertheless the results of the simulations assuming linearity are shown in Figure 6 because the conclusions that were drawn remained unchanged. For rectal temperature, creeping speed, overall clinical lameness score and ground reaction vertical force, a nearly maximal effect of cimicoxib was predicted to occur for doses of 1, 1.5, 2, and 3 mg/kg, respectively. For higher cimicoxib doses, only the duration of the maximum effect was increased. However, considering ground reaction vertical force, the magnitude of the simulated effect increased at doses > 2 mg/kg; a dose of 2 mg/kg was finally selected for confirmatory clinical trial and for that dose, the duration of the different pharmacological effects to mitigate a permanent inflammatory stimulus ranged between 10 to 15 h for rectal temperature, creeping test, vertical ground reaction force and clinical lameness score, with this duration being sufficient to provide satisfactory clinical results (see Discussion).

### Study 2

**Figure 7:**
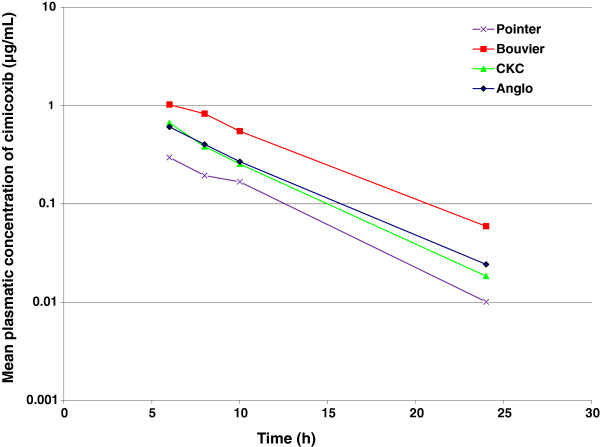
**Mean plasma concentrations (μg/mL) of cimicoxib vs. time in 4 different breeds after an oral administration of cimicoxib at 2 mg/kg.** Mean values for 10 dogs per breed in 4 different breeds (Cavalier King Charles Spaniels, Pointers, Anglo-French Hounds and Bernese Mountain). Visual inspection of the curves suggests differences between breeds likely due to differences in bioavailability.

Figure 7 shows the average terminal slope of cimicoxib after an oral administration of cimicoxib to 10 dogs belonging to four different breeds. The half-life was computed and descriptive statistics are presented by breed in Table 4. Mean values of plasma half-life were 2.7 h, 3.2 h, 3.9 h and 4.4 h for: cavalier King Charles, Anglo-French Hounds, Pointers, Bernes Moutain respectively (P < 0.001, ANOVA). The intra-breed variability of the terminal half-life was similar in each breed, with a coefficient of variation of 20-25%.

**Table 4:** Mean plasma half-life and other descriptive statistics of cimicoxib in 4 dogs breeds after a single oral administration of cimicoxib at 2 mg/kg

	**Anglo-French hounds**	**Pointers**	**Cavalier King Charles spaniels**	**Bernese mountain dogs**
**Number of dogs**	10	10	10	10
**Minimum (h)**	2.0	2.5	1.9	3.2
**Maximum (h)**	4.0	5.9	3.5	5.8
**Mean (h)**	3.2^a,b^	3.9^a,c^	2.7^b^	4.4^c^
**Standard Deviation (h)**	0.7	1	0.6	0.9
**CV (%)**	22.7	26.5	21.6	20.1

^a,b,c^ : The values indexed by the same letter are not significantly different.

## Discussion

Cimicoxib is a new coxib marketed in veterinary medicine and with a terminal half-life between 2 and 4 h; its pharmacokinetic profile is intermediary between that of robenacoxib (half-life of 1 h) and that of firocoxib (half-life of 7 h). During this experiment in beagle dogs we identified two subgroups of subjects: one group of dogs eliminating cimicoxib from plasma at a rapid rate and a second group of dogs having a three-fold lower plasma clearance ; they were termed by others who observed the same phenomenon for celecoxib [23] extensive metabolizers (EM) and poor metabolizers (PM). The origin and mechanism of these different elimination patterns of cimicoxib were not investigated but are likely to be due to polymorphism of the enzymes cytochrome P450 2D15 (CYP2D15) in dogs, which have been shown to be involved in the polymorphism of canine celecoxib metabolism [23]. For mavacoxib, broad variability in pharmacokinetics was also observed within laboratory beagle dogs with a typical half-life of 15–17 days while some of these beagle dogs had a half-life >30 days. For mavacoxib it was postulated that there was polymorphism of a transporter involved in the biliary clearance even if different sub-populations could not be detected [12]. No equivalent information has been published for firocoxib. In the present experiment, in order to select the most relevant subpopulation of beagle dogs (EM or PM) regarding the future target dog population we conducted a pilot study in field conditions on four different dog breeds of different body size (Anglo-French Hounds, Pointers, Cavalier King Charles Spaniels and Bernese Mountain Dogs) and a rather short terminal half-life (from 2-4 h) was observed for the 40 dogs investigated and we decided that our subpopulation of EM beagle dogs should be considered for the PK/PD dosage regimen determination.

For PK/PD investigations, a reversible kaolin paw inflammation model i.e. an ethically acceptable inflammation model was used to determine the main PD parameters of cimicoxib for a set of clinically relevant endpoints able to reflect the antipyretic, analgesic and anti-inflammatory potential. This model has been validated in our hands and is fully described elsewhere [15]. For the current experiment, the duration of the cimicoxib effect was short enough to avoid having to model the time development of inflammation as was required for meloxicam using the same protocol [15]. In other words, it was assumed that the natural progression of the inflammation following a kaolin administration (increasing then decreasing after a more-or-less well-defined plateau phase) was not a relevant confounding factor to assess the cimicoxib effect. In the model for the placebo period, body temperature, creeping time, and paw withdrawal time still were altered in first 56 h after kaolin administration (spontaneous improvement was 50%, a 100% improvement meaning return to the prekaolin administration condition). This model (inflammation >24 h) used to test daily administration will be preferred to model with short inflammation like sodium urate crystal (inflammation <18 h); model used for robenacoxib and firocoxib [24,25]. Using a classical PK/PD modelling approach, the cimicoxib efficacy (expressed as a percentage of return to control values) and the cimicoxib potency (measured by the plasma EC_50_/IC_50_) were determined. For the subgroup of EM, EC_50_/IC_50_ for rectal temperature, creeping speed under a tunnel, vertical ground reaction force and a clinical lameness, scores were between 0.422 and 0.745 nmol/ml *i.e.* slightly lower than those obtained for meloxicam that were between 0.553 and 1.439 nmol/mL using the same canine model and the same investigated endpoints indicating that cimicoxib is slightly more potent than meloxicam, a reference NSAID in dogs. The efficacy parameters *i.e.* Imax were similar for cimicoxib and meloxicam (from 70 to 100% for cimicoxib and from 80 to 100% for meloxicam) indicating that both drugs can totally or near totally suppress symptoms associated with the kaolin inflammation (hyperthermia, lameness).

For the analgesic effect, it was shown that cimicoxib has two types of effect: a so-called anti-hyperalgesic effect (*i.e.* an increase in the pain tolerance threshold which was abnormally lowered by the inflammation) and also an increase to the pain tolerance threshold beyond that recorded during the control (placebo) period suggesting a mechanism of cimicoxib action independent from its anti-inflammatory effect. A similar observation has already been seen with other COXIBs. For example, using a model of peripherally induced inflammatory pain (intraplantar injection of carrageenan) in rats, celecoxib given systemically induced a state of hypoalgesia where the (ipsilateral) nociceptive threshold was raised above basal values. This effect was not observed after treatment with non-selective inhibitors of COX (indomethacin, piroxicam). As naltrexone was able to reverse this hypoalgesia, it was concluded that celecoxib could act centrally *via* an effect mediated by endogenous opioids rather than by inhibition of PG biosynthesis [26]. Recently in rat, it was demonstrated that analgesic central effects of celecoxib were prevented by selective μ-(β-funaltrexamine) and δ-(naltrindole), but not ĸ-(nor-binaltorphimine) opioid antagonists, after intracerebroventricular administration [27]. This genuine effect of COXIBs should deserve attention because cimicoxib could have some advantages over non-COXIB NSAIDs to treat some (chronic) conditions in which pain is unrelated to inflammation.

The ultimate goal of the present experiment was to assist in the selection of a dosage regimen for subsequent assessment in clinical trials. For this purpose, dose-effect relationships were simulated using PK and PD parameters obtained in the EM beagle dogs because their PK parameters were closer to those of the four non-beagle breed dogs. As explained in the result section, our simulations assumed a linear disposition of cimicoxib. However, the disposition of cimicoxib after an oral administration was subsequently shown to be not linear due to solubility saturation and the results of our simulations should only be considered as indicative. Visual examination of the simulated dose-responses (from 0.1 to 8 mg/kg) with the various PD endpoints (rectal temperature, creeping speed to cross a tunnel, vertical ground reaction force of paw and clinical lameness score), showed that a near-maximal effect was obtained with a dose of 2 mg/kg for all effects but the vertical force and that, above this dose, a further increase was only able to increase the duration of the cimicoxib effect. Visual inspection of the PK/PD simulations clearly showed that a full pharmacological effect of cimicoxib cannot be maintained over a 24 h dosage interval with a single daily dose. However, it was considered that to split the daily dose was not in order given that for other short acting COXIBs such as robenacoxib, a once daily administration (1 mg/kg) was shown to be clinically appropriate; Clinical trials with cimicoxib supported this interpretation (see later) and this is likely due to the fact that our experimental model is more severe that most of the clinical conditions encountered and also to the likely longer persistence of the cimicoxib in the synovial fluid of the target dog population as already shown for robenacoxib [28]. Higher concentration of coxib and longer residence times in inflammatory exudate compare to blood is likely a property of class [1,29-31] and it is reasonable to assume that it is also true for cimicoxib. To support this single cimicoxib daily dose, the company carried out field trials on peri-operative pain associated with orthopaedic or soft tissue surgery using carprofen as a comparator (4 mg/kg per day). Carprofen is as well-established NSAID with a half-life of about 9 h in dogs [32], i.e. a half-life well-suited for a single daily dose regimen. The non-inferiority of cimicoxib was concluded in comparison to carprofen on pain during the first 24 hours using a composite pain clinical score and a 20% difference as the inferiority limit [33]. For the relief of pain and inflammation associated with osteoarthritis, the non-inferiority of cimicoxib against firocoxib was also demonstrated with regard to the primary efficacy endpoint and an inferiority limit set to 20% [34]. As cimicoxib administered once a day at a dose of 2 mg/kg was shown not to be inferior to carprofen and firocoxib, two drugs having a longer half-life than cimicoxib, the European Agency concluded that although PK/PD data indicated that the duration of the effect was shorter than the proposed dosing interval, this was regarded as acceptable, given that appropriate information to this effect was included in the product literature.

## Conclusions

Cimicoxib is an efficacious anti-inflammatory and analgesic drug and thanks to a PK/PD approach, a rational dosage regimen of 2 mg/kg was selected for confirmatory clinical trials allowing *in fine* the claim that cimicoxib at a daily dose of 2 mg/kg is not inferior to two well-established comparators, namely carprofen and firocoxib.

## Methods

### Animals

#### Study 1

Twelve healthy Beagle dogs (6 females and 6 males) were selected after clinical examination and biochemical analysis to exclude any underlying pathologies. The bodyweights and ages were 10 ± 2.5 kg and 1 ± 0.25 years, respectively. The dogs were usually housed in large boxes (2 per box). During the different periods of the trial the animals were kept in individual stainless steel cages in a controlled environment. Dogs were fed once a day with 250 g of commercial dry food (Medium, Royal Canin SA – Aimargues – France). On the day of the trial, they were fed on the evening after the last measurements. As the inflammatory model is totally reversible without sequelae, all the dogs that participated in this trial were rehomed as companion animals at the end of the study.

#### Study 2

Four different sized-breeds from four different private breeders were tested (ten individuals were used from each breed): Anglo-French Hounds, Pointers, Cavalier King Charles Spaniels and Bernese Mountain Dogs. The bodyweights of the dogs ranged between [27.2, 37.4], [16.8, 22.3], [7, 9.8], [44.5, 62.5] kg; the ages were [0.6, 2.5], [1.9, 11.8], [1.7, 6.7], [1.4, 6.4] years for the Anglo-French Hounds, Pointers, Cavalier King Charles Spaniels and Bernese Mountain Dogs breeds, respectively. The sex ratios (male/female) were 5/5, 6/4, 4/6 and 4/6 for these same breeds. Prior to the experiment, all the animals underwent a clinical examination by a veterinarian, along with a complete biochemistry profile. No clinically important abnormality was noted during these examinations.

Animal care and conduct of the study were performed according to the Guide for the Care and Use of Laboratory Animals. The protocol was approved by the animal experimentation ethics committee of Midi-Pyrenees (MP/01/41/09/08). The study was performed in compliance with the Principles of Good Clinical Practice (CVMP/VICH/595/98) and according to the Guideline for the Conduct of Efficacy Studies for NSAIDs (EMEA/CVMP/237/01).

### Experimental design

#### Study 1

The 12 dogs were regularly trained to get accustomed to experimental and measurements conditions for at least one month before the beginning of the experiment. Furthermore, all the investigators involved received a special training for endpoints measurements. The paw inflammation model used was the kaolin model (1.55 g of Kaolin aseptically injected in the skin under general anesthesia, [15]). The measured endpoints used to evaluate the antipyretic, analgesic and anti-inflammatory effects of the NSAID were the body temperature, the paw circumference, the time to perform a creeping test under a tunnel, the lameness score, the vertical force of the paw on force plates (normalized to the dog’s body weight), the thermal pain threshold and the plantar skin temperature [15]. For body temperature a single measurement was obtained at each time using an electronic thermometer. Paw circumference duplicate measures per time point was done just above the pad using a measuring tape (DMC, Colmar, France). Skin plantar temperature was measured (in degrees Celsius) using an infrared thermometer (Raynger M. Raytek, Fisher Bioblock Scientific - Illkirch – France) at a reference site on the plantar face of the paw, at six different times for each day and measures were performed in triplicate. Data used was the average of the three measurements performed at each time point. Creeping time was measured (in seconds) in a tunnel 6.38 m long, using a stopwatch (digital stopwatch) in triplicate for each time point and creeping speed (m/s) was used as the final measurement for this endpoint. Lameness score was assessed using a numerical rating scale as developed by Giraudel et al. [35]. Vertical normalized force i.e. the maximal vertical force applied on the ground by a hind limb was measured with force plates (SATEL Veto; Patrick Savet, Blagnac, France); for each measurement time, three measures were recorded, the mean values were normalized to the dog’s body weight (*F*max/b.wt., kg/kg) and used in the data analyses. Pain was assessed using a hind paw thermal escape model. The model consisted of exposing the hind limb to a light beam delivered by a Hargreaves apparatus (model 390; IITC Inc., Woodlands Hills, CA) [13,36]. A heat intensity corresponding to 15% of the peak heating value of 150 W was selected. The paw withdrawal time (in seconds) was measured. For each trial, three measures were recorded per measurement time, and the mean of the three measures was used in the data analysis.

The study was conducted according to a classical 2×2 crossover design. Briefly, in period one, half of the dog (3 males and 3 females), received a single oral dose of cimicoxib (approximately 2 mg/kg, one 20 mg capsules – Vetoquinol Lure france) 26.5 h after the induction of kaolin inflammation on the left paw; the other half dogs received a placebo treatment at the same time. In period 2, treatments were crossed over for each dog and inflammation was induced on the right paw. A wash-out delay of at least 5 weeks was applied between the two periods. The treatment (cimicoxib or placebo) was blinded to the investigators.

Blood samples (5 mL, heparinized tubes) were obtained from the jugular vein by direct puncture just before inflammation to perform a biochemical checkup of the animals at the beginning of the experiment. Time 0 was defined as the time of treatment administration. Blood samples (5 mL, heparinized tubes) were collected at time 0 (just before cimicoxib or placebo administration) and then at times 30, 60, 90 min, and, 4, 6, 9, 15, 30 and 48 hours post-treatment. Tubes were centrifuged at 3000 G and plasma samples were stored at -20°C.

At least three days before the induction of kaolin inflammation, the PD endpoints were measured twice on the same day to generate negative control values. These endpoints were also measured at 23.5 and 25.5 h after the induction of the inflammation (i.e. before the treatment) to generate positive control values. Subsequently, the PD endpoints were measured at 2, 4, 9, 15 hours after administration (measures on all dogs) and at 22 h and 27 h after administration depending on the presence of further cimicoxib activity.

The results of this first study suggested that there might be two subpopulations of dogs diverging in their capacities to eliminate cimicoxib. To verify this hypothesis, a PK investigation of cimicoxib following IV administration (bolus, 2 mg/kg) was performed on the 12 dogs at least 2 months after the completion of the PK/PD study. Blood samples were obtained from the jugular vein by direct puncture at time 0 (control just before the IV administration) and then 2, 15, 30, 60, 90 min. and 2, 3, 4, 6, 9, 12, 24, 30 and 48 hours after the test article administration.

#### Study 2

The half-life of elimination of cimicoxib was studied in four different sized breeds (40 dogs). Two mg/kg of cimicoxib was administered orally, in the form of 10 and 20 mg (hard gelatin capsules, Vetoquinol). For each dog, four blood samples (5 mL, heparinized tubes) were taken, at 6 h, 8 h, 10 h and 24 h after administration of cimicoxib, centrifuged (3000 G) and stored (- 20°C).

### Assay of cimicoxib in plasma

Plasma samples were analyzed by a High Performance Liquid Chromatography (HPLC) method using ultraviolet (UV) detection. Briefly, an internal standard (UR-8877) and cimicoxib (UR-8880) were extracted from plasma by a solid liquid (methanol) extraction process using HLB Oasis cartridges (Waters). The extraction yield was about 90%. The HPLC apparatus consists of a pump system equipped with an automatic injector and an UV detector (242 nm). Separation was achieved by reverse phase column with an octadecylsilane stationary phase (Merck Lichrospher 100 RP18e (125×4) mm, 5 μm) using a guard column (Merck Lichrospher 100 RP18e (4×4) mm, 5 μm). The mobile phase was a mixture of 0.65 L of ultra-pure water and 0.35 L of acetonitrile which was delivered at a flow rate of 1 mL per min. Under these conditions, cimicoxib (UR-8880) and the internal standard (UR-8877) were eluted at retention times of 6 to 7 min and 10 to 11 min, respectively. The method was linear over the calibration range of 0.01 to 5 μg/mL using a linear model weighted by 1/X^2^. Within-day and day-to-day coefficients of variation were less than 9% and the accuracy ranged from 94 to 103%, indicating an appropriate precision and accuracy for the analytical method. The lack of interference from endogenous compounds was verified on blank plasma from untreated dogs, establishing the specificity of the method. The validated limit of quantification was 0.01 μg/mL.

### Data analysis and modeling

#### Study 1

Pharmacokinetic and PK/PD modeling were performed by least-squares regression analysis using WinNonlin Professional software (WinNonlin® software, version 4.0.1, Pharsight Corporation, Mountain View, Ca, USA). For PK analysis, individual plasma cimicoxib concentrations were fitted to polyexponential equations. The data points were weighted by the inverse of the squared-fitted value. The number of exponential (2 or 3) terms needed to obtain the best fit for each data set was determined by the Akaike’s information criteria [37] and by inspecting the plot of residuals. A biexponential equation corresponding to a mono-compartmental model for extravascular administration with a lag-time was selected (Equation 1).

(1) $$C\left(t\right)=\frac{\text{FD}k_{01}}{V\left(k_{01}-k_{10}\right)}\left[\exp\left(-k_{10}\times \left(t-t_{\text{lag}}\right)\right)-\exp(-k_{01}\times \left(t-t_{\text{lag}}\right)\right]$$

where C(t) is the cimicoxib plasma concentration (μg/L) at time t (h), V/F (L/kg) is the apparent volume of distribution, k01 (1/h) is the rate constant of the initial ascending phase, k10 (1/h) the rate constant of the terminal phase, tlag is the lag time and D is the cimicoxib dose (mg/kg). The parameters (V/F, k01 and k10 and tlag) were estimated.

For the IV study, a non-compartmental analysis was used to determined PK parameters.

For PD analysis, a percentage of improvement was calculated for all endpoints (except for the lameness score) following Equation 2:

(2) $$\%\;\text{improvement}\;=\left(\frac{T^{+}-M}{T^{+}-T^{-}}\right)*100$$

Where M is the measurement of the endpoint at different times after administration of cimicoxib, T^-^ is the mean negative control value (before inflammation) and T^+^ is the mean positive control value (after inflammation but before any treatment).

The maximum effect was defined as the mean % of improvement for a duration corresponding to a plateau of effect as identified by the visual inspection of the graph representing the % of improvement vs. time.

As the effect cimicoxib on the local temperature and paw oedema after inflammation were present but lower than the other effects, these endpoints were not used for the PK/PD analysis. Similarly the analgesic effect was discarded for modelling because it appeared to follow a complicate pattern suggesting a dual mechanism of action, namely an anti-nociceptive phenomenon and anti-hyperalgesic one (see Discussion), requiring a more advanced modelling with appropriate data that were not available in this experiment. Finally, four endpoints were considered as suitable for the PK/PD analysis aiming at modelling the relationship between cimicoxib plasma concentrations and its antipyretic and anti-inflammatory effects, namely: body temperature (antipyretic effect), creeping speed, vertical force of the paw and clinical lameness score (anti-inflammatory effects).

The selected PK/PD models were of the class of the indirect response models as proposed by Dayneka [38]. In these models, the measured response (R) is assumed to result from factors controlling either the development (Kin) or the dissipation (Kout) of the response according to Equation 3.

(3) dRdt=Kin-Kout×R

where dR/dt is the rate of change of the response over time, Kin represents the zero-order rate constant for production of the response and Kout the first-order rate constant for loss of the response. The time development of measured response was modeled by a sigmoid-Emax (maximum stimulatory effect) or sigmoid-Imax (maximum inhibition effect) function (Hill models) that can be exerted either on Kin or Kout. The parameters generated by such analyses establishing the relationship between C(t), the plasma drug concentration at time *t*, and the effect, are the “Emax” or “Imax” for the drug effect (estimated maximum indirect effect), the “EC_50_” or “IC_50_” for the potency (estimated plasma concentration which causes 50% of Emax or Imax), and the Hill’s coefficient (n) for the sensitivity of the different concentration-effect curves. The effect of cimicoxib on the central temperature was based on a model assuming that during the inflammation phase, thermogenesis is increased (increase in Kin) while thermolysis is reduced (reduction in Kout) until a new balance for central temperature is reached. The antipyretic effect of an NSAID consisted of an increase in thermolysis (increase of Kout) enabling the central temperature to return to its control values, according to Equation 4:

(4) $$\frac{\mathrm{dR}}{\mathrm{dT}}=\text{Kin}-\text{Kout}\times \left(1+\left(\frac{E\max\times C^{n}}{E{C_{50}}^{n}+C^{n}}\right)\right)\times R$$

For the creeping test consisting of crossing the tunnel more or less rapidly, an empirical model postulating that the dog’s speed (m.s^-1^) is the result of a balance between its motivation to move forward (Kin) and a braking effect (Kout) associated with the severity of inflammation with a lower Kout in control conditions than during inflammation. During inflammation, pain increases the braking effect (i.e. increase Kout) and decreases the speed to cross the tunnel. The anti-inflammatory effect of cimicoxib consisted in reducing the pain (by decreasing Kout), which in turn increased the speed to cross the tunnel as described by Equation 5:

(5) dRdT=Kin-Kout×1-Imax×CnIC50n+Cn×R

For the vertical force measured with the force plate apparatus normalized by the weight of the dog (without units), Kin represents the ground reaction forces in control resting conditions and Kout represents the factors prompting the withdrawal of limb from the ground in the case of inflammation due to pain *i.e.* that Kout is increased in the case of inflammation. Thus, the anti-inflammatory effect of cimicoxib consisted in a reduction of pain (decrease of Kout) and the same model as for the creeping speed under the tunnel was used (see Equation 5).

For the clinical lameness score, an empirical model was used postulating that the clinical score, from 0 (dog without lameness) to 5 (dog does not put limb on the ground), was the result of a balance between factors promoting lameness (Kin) and factors mitigating lameness (Kout). During inflammation, Kin is increased and the effect of cimicoxib was modelled using a concentration-dependent inhibition of Kin, according to Equation 6:

(6) $$\frac{\mathrm{dR}}{\mathrm{dT}}=\text{Kin}\times \left(1-\left(\frac{C^{n}}{I{C_{50}}^{n}+C^{n}}\right)\right)-\text{Kout}\times R$$

#### Study 2

Half-life was estimated by log-linear regression (estimating the slope) using the non-compartmental approach implemented in Winnonlin.

### Statistical analysis

Statistical analysis was carried out using Systat (Version 8.0, SPS Onc., Chicago, IL). For study 1, the comparison between the two identified dog subpopulations for cimicoxib was done with an unpaired t-test. For study 2, the effect of the breed on cimicoxib plasma half-life was analyzed with a one-way ANOVA with the breed as fixed effect factor.

## Abbreviations

NSAID: Non-steroidal anti inflammatory drug; IV: Intravenous; SM: Poor metabolizers; EM: Extensive metabolizers; PK/PD: Pharmacokinetic/Pharmacodynamic; COX: Cyclooxygenase; g.i.t: gastrointestinal tract; CYP: Cytochrome; HPLC: High performance liquid chromatography; UV: Ultra violet.

## Competing interests

This study was sponsored by Vetoquinol and the charges of the article will be paid by Vetoquinol.Schneider M, and Woehrle F are employed by Vetoquinol.

## Authors’ contributions

Contributions, Study 1 (PK/PD study): Project design: PLT, EJ, MS, FW. Management: PLT; Writing of the project: PLT, EJ, MS, FW; Animal phase: EJ; PK analysis: PLT, EJ; PD analysis and simulation: PLT, EJ; Statistical analysis: EJ; Writing of the paper: PLT, EJ,MS,FW. Study 2 (IV study) Project design: HL, MS, FW; Management: HL; Writing of the project: HL, MF, EJ, MS, FW; Animal phase: MF, EJ; PK analysis: HL, MF; Statistical analysis: HL, MF; Writing of the paper: PLT, EJ, MS, FW. All authors read and approved the final manuscript
